# Supplementary material for: Miraculous Al/PDF Composites Using NF2 to Enhance the Energy Release of Al, Prepared Through an Efficient Method
Source: Nanomaterials (Basel). 2024 Dec 10;14(24):1980. doi: 10.3390/nano14241980 (PMC11676450; doi:10.3390/nano14241980)
Supplement: Supplementary file 1 [file nanomaterials-14-01980-s001.zip › nanomaterials-3330855-supplementary.pdf]

Supporting Information
for
Miraculous Al/PDF Composites, Through NF_2 to Enhance the Energy Release of Al, Prepared Through an Efficient Method

Junqi He [1,2], Jing Lv [2], Wenfang Zheng [1,2,*], Renming Pan [1,2] and Yanan Li [1,2]

[1] School of Safety Science and Engineering (School of Emergency Management), Nanjing University of Science and Technology, Nanjing 210094, China

[2] School of Chemistry and Chemical Engineering, Nanjing University of Science and Technology, Nanjing 210094, China

* Correspondence: zhwf@njust.edu.cn

Contents

Figure S1. Statistics of ignition combustion data: (a) ignition delay of Al and Al/PDF; (b) combustion duration of Al and Al/PDF; (c) ignition delay of mix samples(AP and Al or Al/PDF); (d) combustion duration of mix samples(AP and Al or Al/PDF).

Figure S2. Chromatograms for AP and Al/PDF at 300 °C.

Figure S3. Chromatograms for AP and Al/PDF at 500 °C.

Table S1. The main chemical compounds of AP and Al/PDF (29 μm) by PY/GC-MS.

Table S2. The main chemical compounds of AP and Al/PDF (13 μm) by PY/GC-MS.

Table S3. The main chemical compounds of AP and Al/PDF (1~3 μm) by PY/GC-MS.

Figure S4. EDS of collected combustion residues of mixtures of AP : (a) Al (29 μm); (b) Al (13 μm); (c) Al (1~3 μm); (d) Al/PDF (29 μm); (e) Al/PDF (13 μm); (f) Al/PDF (1~3 μm).

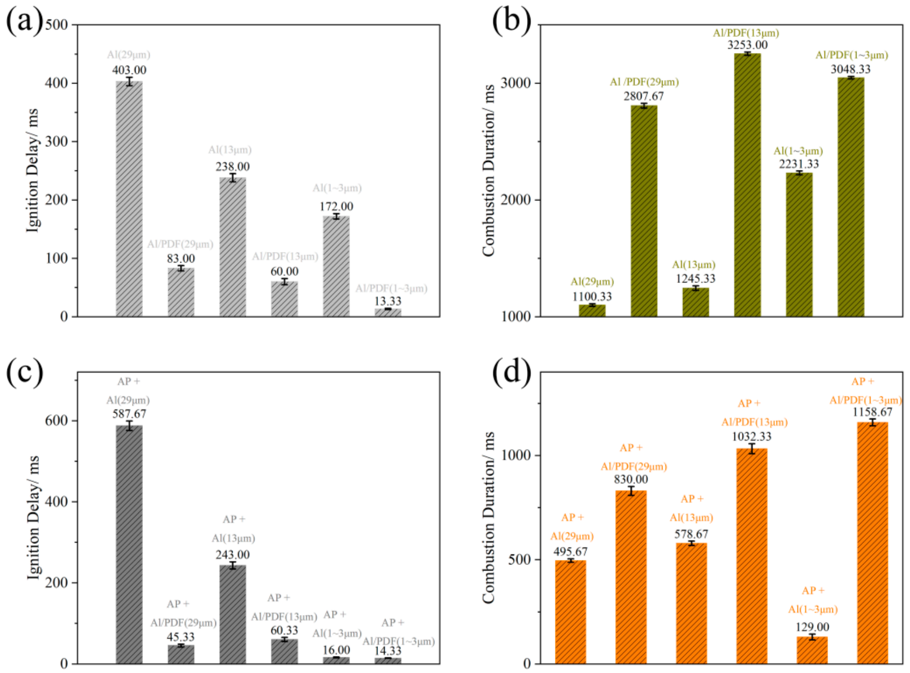

Figure S1. Statistics of ignition combustion data: (a) ignition delay of Al and Al/PDF; (b) combustion duration of Al and Al/PDF; (c) ignition delay of mix samples(AP and Al or Al/PDF); (d) combustion duration of mix samples(AP and Al or Al/PDF).

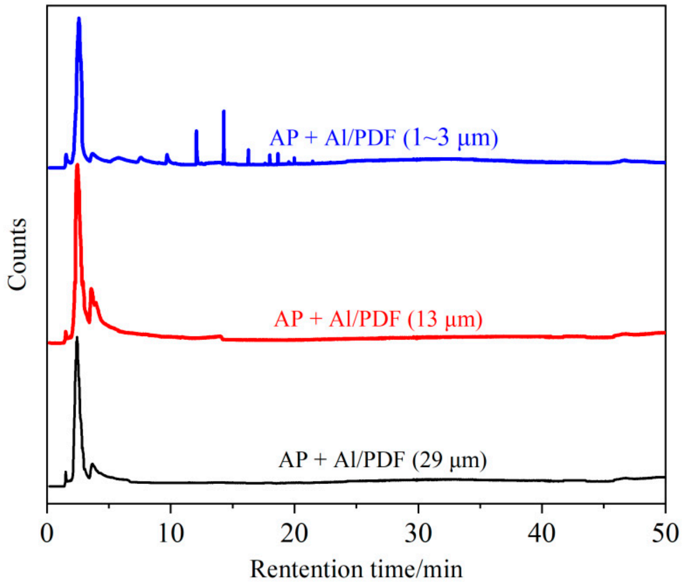

Figure S2. Chromatograms for AP and Al/PDF at 300 °C.

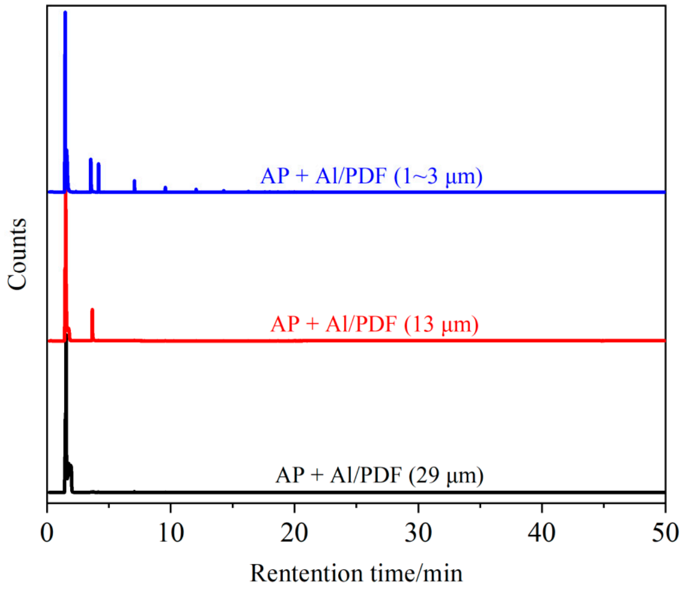

Figure S2. Chromatograms for AP and Al/PDF at 500 °C.

Table S1. The main chemical compounds of AP and Al/PDF (29 μm) by PY/GC-MS

Temperature/°C	Retention time/min	Compound	Chemical formula
300	2.432	*N*-Methylethylenediamine	$C_3H_{12}N_2$
	3.677	Glyoxylic acid	$C_2H_2O_3$
	13.915	2-Hexenedioic acid, 2,4-dichloro-5-oxo-	$C_6H_4Cl_2O_5$
500	1.423	Aminoacetic acid	$C_2H_5NO_2$
	1.579	Alanine	$C_3H_7NO_2$
	1.747	3-Amino-2-methylpropanoic acid	$C_4H_9NO_2$

Table S2. The main chemical compounds of AP and Al/PDF (13 μm) by PY/GC-MS

Temperature/°C	Retention time/min	Compound	Chemical formula
300	2.458	*N*-Methylethylenediamine	$C_3H_{12}N_2$
	3.568	Glyoxylic acid	$C_2H_2O_3$
	13.915	Ammonium chloride	NH_4Cl
500	1.518	Aminoacetic acid	$C_2H_5NO_2$
	1.684	Alanine	$C_3H_7NO_2$
	1.762	3-Amino-2-methylpropanoic acid	$C_4H_9NO_2$
	3.657	Fluoro(trinitro)methane	CFN_3O_6
	7.060	Dimethylphenylfluorosilane	$C_8H_{11}FSi$

Table S3. The main chemical compounds of AP and Al/PDF (1~3 μm) by PY/GC-MS

Temperature/°C	Retention time/min	Compound	Chemical formula
300	2.589	N-Methylethylenediamine	$C_3H_{12}N_2$
	3.746	Glyoxylic acid	$C_2H_2O_3$
	5.773	Chlorobenzene	C_6H_5Cl
	9.705	*N*-(Trifluoroacetyl)-*N,O,O',O''*-tetrakis(trimethylsilyl)norepinephrine	$C_{24}H_{34}F_5NO_3Si_3$
	12.071	Dodecamethylcyclohexasiloxane	$C_{12}H_{36}O_6Si_6$
	16.265	*N*-[(Pentafluorophenyl)methylene]-β,3,4-tris(trimethylsilyloxy)benzeneethanamine	$C_{24}H_{34}F_5NO_3Si_3$
	17.611	4-(3,5-Dimethylphenyl)phenylacetic acid	$C_{25}H_{24}O_4$
	18.000	Octadecamethylcyclononasiloxane	$C_{18}H_{54}O_9Si_9$
	18.667	Tetradecamethylheptasiloxane	$C_{14}H_{44}O_6Si_7$
	19.988	(4-methoxy-2,6-dimethylphenyl)-(4-nitrophenyl)diazene	$C_{15}H_{15}N_3O_3$
500	1.449	Aminoacetic acid	$C_2H_5NO_2$
	1.466	Alanine	$C_3H_7NO_2$
	1.579	3-Amino-2-methylpropanoic acid	$C_4H_9NO_2$
	3.540	Fluoro(trinitro)methane	CFN_3O_6
	4.166	Dimethylphenylfluorosilane	$C_8H_{11}FSi$
	7.068	Octamethyl cyclotetrasiloxane	$C_8H_{24}O_4Si_4$
	9.566	Benzyltriethoxysilane	$C_{13}H_{22}O_3Si$
	12.045	Dodecamethylcyclohexasiloxane	$C_{12}H_{36}O_6Si_6$
	14.280	Tetradecamethylcycloheptasiloxane	$C_{14}H_{42}O_7Si_7$
	16.274	1,1,3,3,5,5,7,7,9,9,11,11,13,13-Tetradecamethylheptasiloxane	$C_{14}H_{44}O_6Si_7$
	18.003	Dodecamethylcyclohexasiloxane	$C_{12}H_{36}O_6Si_6$
	18.669	1-Naphthalenol,4-[2-(4-nitrophenyl)diazenyl]-	$C_{16}H_{11}N_3O_3$
	19.991	Tetrabenzo[*e,i,o,s*][1,4,7,11,14,18]dioxatetraazacycloeicosine,6,7,19,20,21,22-hexahydro-	$C_{30}H_{32}N_4O_2$
	20.963	*N*-(Trifluoroacetyl)-*N'*-tetrakis(trimethylsilyl)norepinephrine	$C_{22}H_{42}F_3NO_4Si_4$

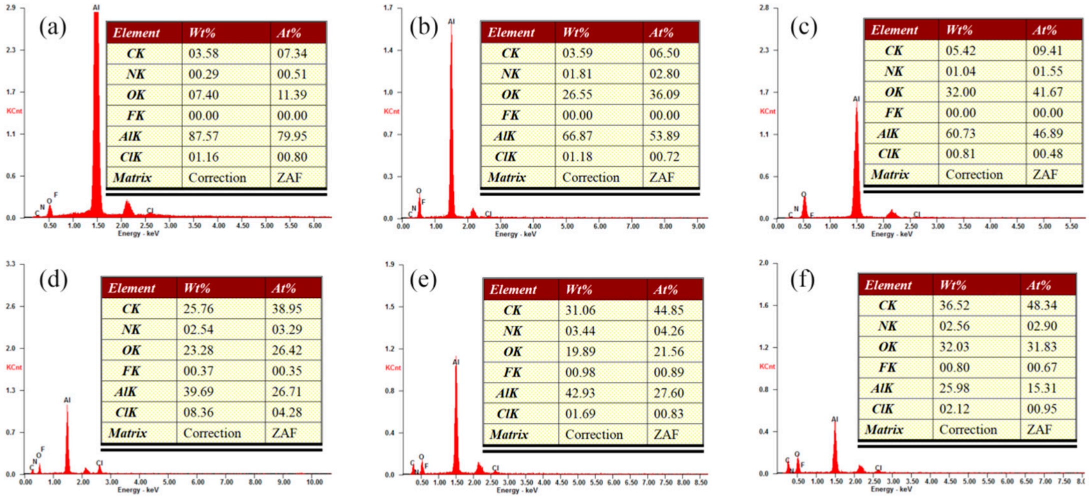

(a)

Element	Wt%	At%
CK	03.58	07.34
NK	00.29	00.51
OK	07.40	11.39
FK	00.00	00.00
AlK	87.57	79.95
ClK	01.16	00.80
Matrix	Correction	ZAF

(b)

Element	Wt%	At%
CK	03.59	06.50
NK	01.81	02.80
OK	26.55	36.09
FK	00.00	00.00
AlK	66.87	53.89
ClK	01.18	00.72
Matrix	Correction	ZAF

(c)

Element	Wt%	At%
CK	05.42	09.41
NK	01.04	01.55
OK	32.00	41.67
FK	00.00	00.00
AlK	60.73	46.89
ClK	00.81	00.48
Matrix	Correction	ZAF

(d)

Element	Wt%	At%
CK	25.76	38.95
NK	02.54	03.29
OK	23.28	26.42
FK	00.37	00.35
AlK	39.69	26.71
ClK	08.36	04.28
Matrix	Correction	ZAF

(e)

Element	Wt%	At%
CK	31.06	44.85
NK	03.44	04.26
OK	19.89	21.56
FK	00.98	00.89
AlK	42.93	27.60
ClK	01.69	00.83
Matrix	Correction	ZAF

(f)

Element	Wt%	At%
CK	36.52	48.34
NK	02.56	02.90
OK	32.03	31.83
FK	00.80	00.67
AlK	25.98	15.31
ClK	02.12	00.95
Matrix	Correction	ZAF

Figure S4. EDS of collected combustion residues of mixtures of AP : (a) Al (29 µm); (b) Al (13 µm); (c) Al (1~3 µm); (d) Al/PDF (29 µm); (e) Al/PDF (13 µm); (f) Al/PDF (1~3 µm).
